# A fault diagnosis method for wireless sensor network nodes based on a belief rule base with adaptive attribute weights

**DOI:** 10.1038/s41598-024-54589-6

**Published:** 2024-02-19

**Authors:** Ke-Xin Shi, Shi-Ming Li, Guo-Wen Sun, Zhi-Chao Feng, Wei He

**Affiliations:** 1 Harbin Normal University, Harbin, 150025 China; 2 Rocket Force University of Engineering, Xi’an, 710025 China

**Keywords:** Wireless sensor network, Fault diagnosis, Belief rule base, Adaptive attribute weights, Engineering, Mathematics and computing

## Abstract

Due to the harsh operating environment and ultralong operating hours of wireless sensor networks (WSNs), node failures are inevitable. Ensuring the reliability of the data collected by the WSN necessitates the utmost importance of diagnosing faults in nodes within the WSN. Typically, the initial step in the fault diagnosis of WSN nodes involves extracting numerical features from neighboring nodes. A solitary data feature is often assigned a high weight, resulting in the failure to effectively distinguish between all types of faults. Therefore, this study introduces an enhanced variant of the traditional belief rule base (BRB), called the belief rule base with adaptive attribute weights (BRB-AAW). First, the data features are extracted as input attributes for the model. Second, a fault diagnosis model for WSN nodes, incorporating BRB-AAW, is established by integrating parameters initialized by expert knowledge with the extracted data features. Third, to optimize the model's initial parameters, the projection covariance matrix adaptive evolution strategy (P-CMA-ES) algorithm is employed. Finally, a comprehensive case study is designed to verify the accuracy and effectiveness of the proposed method. The results of the case study indicate that compared with the traditional BRB method, the accuracy of the proposed model in WSN node fault diagnosis is significantly improved.

## Introduction

Wireless sensor networks (WSNs) are commonly used in industrial practices for environmental detection due to their low power consumption and high sensitivity. However, due to the complex and variable working environment of sensor nodes, the failure of sensor nodes is inevitable. Hence, it is crucial to diagnose faults in WSN nodes to ensure industrial production safety and timely data collection^1^.

The commonly used methods for fault diagnosis of WSN nodes are model-based methods, data-driven methods, and hybrid information-based methods^2,3^. The modelling analysis approach realizes fault diagnosis through mathematical mechanisms and functions^4–7^, which is the most frequently used method. However, the accuracy of this method is limited due to the high complexity of the actual environment system. The data-driven method has higher model accuracy and relies on analysing data samples^8^. However, in the harsh environment where the sensors are located, the existence of interference factors will lead to the unreliable data involved in training, which will lead to a reduction in the diagnostic accuracy^9^. The hybrid information-based methods can realize the combination of different methods^10^. This type of approach integrates qualitative knowledge and quantitative data through the use of various models. However, there is a common problem with both model-based and data-driven approaches. It is difficult to ensure the effectiveness and accuracy of the model in different environments^11^.

To solve the above problems, this paper proposes a WSN node fault diagnosis model based on BRB-AAW, where AAW denotes adaptive attribute weights. The main contributions of this paper are listed as follows:

1. A new belief rule base with adaptive attribute weights (BRB-AAW) model is constructed, and the concept of adaptive attribute weights is proposed. The original static attribute weights are improved, and reasonable attribute weights are assigned to all rules, thus improving the performance of BRB in the presence of unreliable data.
2. A WSN fault diagnosis method based on BRB-AAW is proposed to obtain more reasonable attribute weights under different conditions, which can effectively improve the accuracy of the diagnosis results.

The article follows the following structure. "Introduction" provides an overview of the current state of research on node fault diagnosis in WSNs, highlighting the benefits of the approach proposed in this paper. "Related work" describes the popular WSN node fault diagnosis methods and compares them with the BRB-AAW-based diagnosis methodology approach. "Problem formulation" addresses the challenges experienced in WSN node fault diagnosis and presents the underlying model architecture. "Establishment of the WSN nodes fault diagnosis model based on BRB-AAW" elaborates on the extraction process of data features, the model inference process, and the approach used for parameter optimization. In "Case study", the proposed fault diagnosis method is validated through the examination of the specific case. In "Conclusion", the proposed fault diagnosis methods and outlined potential future study directions are summarized.

## Related work

Because of the expanding range of WSN applications, an increasing number of scholars have taken the fault diagnosis of WSN nodes as a research topic^10,11^. Abdalzaher et al. proposed a method for estimating missing sensor data^12^. Mohamed et al. worked through two attack defense methods based on a Stackelberg game to protect sensor nodes from attacks^13^. Seddik et al. presented an improved Stackelberg game method that can detect damaged sensor transmission data more efficiently^14^. Selvakumar et al. provided a trust estimation model based on a fuzzy expert system to evaluate nodes and predict possible future changes based on inference mechanisms^15^. Ullah et al. proposed a novel data aggregation scheme based on node clustering and extreme learning machines that effectively reduces redundant and erroneous data^9^. Laiou et al. used a machine learning-based decision tree algorithm to detect and diagnose faults to detect and classify fault data from WSNs^16^.

Most of the above methods are model-based methods and data-driven methods, so these diagnostic methods have common disadvantages. First, all of these methods require a large and uniform number of samples for training parameters. Second, these methods all set many parameters that have no physical meaning, leading to their low interpretability.

Using a hybrid information-based approach, the belief rule base (BRB) has emerged as a potent method for effectively modelling intricate systems. In 2006, a belief rule-based reasoning method known as RIMER was introduced by Yang et al., employing an evidential reasoning (ER) approach^17^. The utilization of an expert system composed of BRB and ER rules allows for enhanced flexibility in representing a multitude of uncertain information, enveloping vagueness, unpredictability, and ignorance. The determination of parameters within the BRB is entrusted to domain experts, drawing upon empirical knowledge and imbuing them with substantial physical significance within the model. This distinctive characteristic empowers the BRB to attain precise outcomes while relying on minimal training data. Expanding upon these advancements, Zhang et al. presented a method based on wavelet packets and BRB^18^. Nevertheless, it is important to note that this approach still has limitations when dealing with fault data that is not entirely reliable.

In 2020, a fault diagnosis method utilizing a belief rule base with mixed reliability was presented by Cheng et al.^19^. This method specifically considers two disruptive factors that impact the observed data: sensor performance and external environmental influence. These factors are quantified as static reliability and dynamic reliability attributes within the belief rule base (BRB)^20,21^. However, it is worth noting that the current approach for calculating hybrid reliability may not be suitable for input data characterized by substantial variations. In 2022, Sun et al. proposed the BRB fault diagnosis model with an adaptive quality factor (BRB-SAQF)^21^. The method adds the attribute quality factor as a new input attribute to reduce the impact of unreliable data features on fault diagnosis accuracy. Nevertheless, it is notable that the attribute quality factor approach does not take into account the needed changes in attribute weights for different environments.

It is necessary to design a method to mitigate the effect of inaccurate data on the identification process and address the limitation in calculating the reliability of static attribute weights^19^, and a BRB-AAW-based fault diagnosis model for WSN nodes is proposed. First, the concept of adaptive attribute weights is proposed, which can distinguish fault types more effectively. Second, the calculation method of adaptive attribute weights is redesigned on the basis of the static attribute weight calculation method. In addition, the proposed method requires fewer training samples than the neural network method due to the advantage of BRB in utilizing small samples for training^22^. Finally, because the parameters in BRB are established by experts and possess physical significance, this methodology offers a higher level of interpretability compared to neural network approaches. To demonstrate that BRB-AAW can effectively improve the accuracy of diagnostic results, it is compared with popular algorithms that can be used for binary classification.

## Problem formulation

In the following section, the issues in the diagnosis of WSNs are identified, and the structure of the model is constructed based on these issues. The notation dictionary is described in "Dictionary of notations", the basic BRB composition is documented in "Basic belief rule base", and the problem is formulated by the formula in "Problem formulation".

### Dictionary of notations

The notations dictionary, which encompasses the symbols used throughout this article, is provided for clarity and comprehension, as shown in Table 1.

**Table 1:** Dictionary of notations.

Notation	Meaning
$R_{k}$	The $k{\text{th}}$ belief rule
$W$	Number of input attributes in the fault diagnosis model
$a_{1} ,...,a_{W}$	Total $W$ input attributes
${\text{F}}_{1} ,...,{\text{F}}_{W}$	Reference values corresponding to the $W$ input attributes
$H_{1} ,...,H_{N}$	$N$ possible troubleshooting results
$D_{1} ,...,D_{N}$	The belief degree associated with each outcome under the $k{\text{th}}$ belief rule
$\theta_{k}$	Rule weights for the $k{\text{th}}$ belief rule
$\updelta _{1}^{{}} {,}...\updelta _{w}^{{}}$	Attribute weights of the ${\text{W}}$ input attributes
${\text{a}}_{1} \left( t \right){\text{,a}}_{2} \left( t \right)...,{\text{a}}_{w} \left( t \right)$	Data features of ${\text{W}}$ attributes extracted in a time interval
$\mu ()$	Computational function for extracting features from the raw sensor data obtained from WSN
$\Lambda$	Parameters involved in the process of extracting data features
$X$	Raw data collected by the sensor over a period of time interval
$t$	At a certain point in the time interval
$\delta_{{\text{j}}}^{{\text{i}}}$	Adaptive attribute weights (BRB-AAW)
$K$	The total number of belief rules in the model
$\Xi$	Expert knowledge for initializing adaptive attribute weights
$g()$	Calculation function of adaptive attribute weights
$S\left( t \right)$	Predictive fault states for troubleshooting systems
$\tau$	Other parameters involved in the fault result diagnosis function
$f()$	Calculation function of fault diagnosis results
$\tau_{{{\text{best}}}}$	Optimized parameters after optimization algorithm
$\beta$	Parameters in the optimization algorithm
$h()$	Parameter optimization algorithm
$Q$	A time point of the node being diagnosed
$T$	Length of time
$\overline{{\text{a}}}$	The average of the collected data features over the specified time interval
$\upalpha ^{4}$	The standard deviation of the collected data features over the specified Time interval
$\uprho _{{\text{i}}}^{{\text{j}}}$	The degree of matching of the $i{\text{th}}$ attribute in the $j{\text{th}}$ reference value
${\text{F}}_{{\text{i}}}^{{\text{k}}}$	the ${\text{ith}}$ attribute's $k{\text{th}}$ reference value
$\rho_{{\text{k}}}^{{\text{i}}}$	the match of the $i{\text{th}}$ attribute in the $k{\text{th}}$ belief rule
*θ*_*i*_	Weight of the belief rule $i$
$\mho_{i}$	The activation weight of the $i{\text{th}}$ belief rule
**N**	The framework for identifying fault diagnosis models includes $N$ levels
$D_{j,i}$	Belief degree of the $j{\text{th}}$ fault diagnosis result in the $i{\text{th}}$ rule
$p\left( {H_{i} } \right)$	The utility of the $i{\text{th}}$ fault diagnosis
**g**	The $g{\text{th}}$ generation optimization algorithm iteration
$\tau_{{\text{i}}}^{{{\text{g + }}1}}$	The optimized parameters of the $i{\text{th}}$ group in the $(g + 1){\text{th}}$ generation
$\tau_{{{\text{best}}}}^{{\text{g}}}$	Average value of optimized parameters in the $g{\text{th}}$ generation
$\varepsilon_{{}}^{{\text{g}}}$	Step size of the $g{\text{th}}$ generation
${\mathbb{R}}$	Normal distribution
$C_{{}}^{{\text{g}}}$	Covariance matrix of the $g{\text{th}}$ generation
$\lambda$	Number of offspring iterated
$E_{{\text{e}}}$	Parameter vector
${\text{n}}_{{\text{e}}}$	Restricted variables in $\tau_{{\text{i}}}^{{\text{g + 1}}}$
$j$	The number of restricted variables in $\tau_{{\text{i}}}^{{\text{g + 1}}}$
${\text{h}}_{{\text{i}}}$	Weighting factor
$\tau_{{{\text{i:}}\lambda }}^{{\text{g + 1}}}$	The $i{\text{th}}$ solution in the $\left( {g + 1} \right){\text{th}}$ generation of the total $\lambda$ group of optimization parameters
$\sigma$	Number of solutions in the offspring

### Basic belief rule base

The BaseBRB effectively harnesses engineering experience and expert knowledge, showcasing its capability to integrate limited samples of monitoring data and possessing robust nonlinear modelling capabilities. It operates as a modelling approach rooted in IF–THEN rules, comprised of a series of belief rules. The belief rules can be described by Eq. (1).

1 $$\begin{gathered} R_{k} :if{\kern 1pt} a_{1} \left( t \right){\kern 1pt} is{\kern 1pt} \, F_{1} {\kern 1pt} \cap {\kern 1pt} {\kern 1pt} a_{2} \left( t \right){\kern 1pt} is{\kern 1pt} \, F_{2} {\kern 1pt} \cap ... \cap a_{W} \left( t \right){\kern 1pt} is{\kern 1pt} \, F_{W} {\kern 1pt} \, \hfill \\ Then{\kern 1pt} {\kern 1pt} {\kern 1pt} result{\kern 1pt} {\kern 1pt} is{\kern 1pt} \left\{ {\left( {H_{1} ,D_{1} } \right),\left( {H_{2} ,D_{2} } \right),...,\left( {H_{N} ,D_{N} } \right)} \right\} \hfill \\ with{\kern 1pt} {\kern 1pt} rule{\kern 1pt} {\kern 1pt} weight{\kern 1pt} {\kern 1pt} \theta_{k} \hfill \\ and{\kern 1pt} {\kern 1pt} attribute{\kern 1pt} {\kern 1pt} weight{\kern 1pt} {\kern 1pt} \delta_{1}^{{}} ,\delta_{2}^{{}} ...\delta_{w}^{{}} \hfill \\ \end{gathered}$$

In the expression of belief rules, $R_{k}$ denotes the $W{\text{th}}$ belief rule, $a_{1} \left( t \right),...,a_{W} \left( t \right)$ represents a sample with ${\text{W}}$ prerequisite attributes, ${\text{F}}_{1} ,...,{\text{F}}_{W}$ represents the reference value corresponding to the W prerequisite attributes, $H_{1} ,...,H_{N}$ represents the $N$ possible outcomes, $D_{1} ,...,D_{N}$ represents the belief degrees associated with each outcome under the ${\text{k - th}}$ belief rule, $\theta_{1} ,...,\theta_{k}$ denotes the rule weight of the $k{\text{th}}$ belief rule, and $\updelta _{1}^{{}} {,}...,\updelta _{w}^{{}}$ represents the attribute weight of each prerequisite attribute.

### Problem formulation

A typical WSN consists of four main components: the sensor nodes, the wireless transmission channels, the sink node, and the information processing center. The wireless sensor nodes are responsible for collecting various types of environmental data. The wireless transmission channels facilitate communication between different nodes. The sink node detects connections between the region and external networks. Finally, the processing center aggregates and processes data sent by different sensors.

Fault diagnosis can be divided into four parts: obtaining characteristics of faulty sensor data, determining adaptive attribute weights based on different environments, diagnosing faults by combining various information sources, and improving model parameters using optimization algorithms. The four problems are described as follows:

Problem 1: The fault diagnosis model necessitates extracting diverse data features as input attributes. In the realm of WSNs, data obtained from distinct sensors exhibit resemblances, encompassing temporal and spatial correlations. Upon the failure of a WSN node, these correlation features undergo alterations. Hence, it becomes imperative to scrutinize the raw data collected by the sensors and extract data features that bear time-based or spatial associations. These extracted features are employed as input variables. The extraction of data features can be described by Eq. (2).

2 $${\text{a}}_{1} \left( t \right){\text{,a}}_{2} \left( t \right),...,{\text{a}}_{w} (t) = \mu ({\text{X}},\Lambda )$$

The function $\mu ()$ is employed to denote the extraction of features from the raw data acquired by the WSN, where $\Lambda$ signifies the parameters involved in this process. Specifically, ${\text{a}}_{1} \left( t \right){\text{,a}}_{2} \left( t \right),...,{\text{a}}_{w} \left( t \right)$ represents the ${\text{w}}$ attribute data features over a given time interval, while $X$ denotes the raw data gathered by the sensor device within that time duration. The variable ${\text{t}}$ refers to a specific moment within the time interval.

Problem 2: The model is designed to improve the reliability of fixed attribute weights and initialize adaptive attribute weights on its basis. The reliability of the information collected by WSN nodes is affected by the harsh working environment, which leads to deviation in the input attributes and thus affects the accuracy of the attribute weights. In addition, when the diagnostic model is too complex, the update time of the model parameters is long, and the timeliness of its modelled attribute weights is limited. Therefore, expert knowledge is needed to construct an accurate diagnostic model. The process of building adaptive attribute weights can be expressed as Eq. (3).

3 $$\delta_{{\text{j}}}^{{\text{i}}} = {\text{g}}(\delta_{{\text{j}}}^{{}} ,\Xi ),\quad i = 1,...,K,j = 1,...,W$$

$\delta_{{\text{j}}}^{{\text{i}}}$ is the adaptive attribute weight, and K represents the total number of rules in the BRB model.$\delta_{{\text{j}}}^{{}}$ represents the previous fixed attribute weight, $\Xi$ represents the expert knowledge used to initialize the adaptive attribute weight, and $g()$ indicates the function for calculating the adaptive attribute weight.

Problem 3: The fault diagnosis model needs to combine multiple sources of information to reason about the diagnosis results. Restricted by the monitoring environment of WSNs, a large amount of high-value data cannot be collected. The accuracy of models constructed solely from expert knowledge is insufficient. Therefore, it is necessary to consider how the different information collected can be aggregated into the following fault diagnosis model. This BRB inference process with adaptive attribute weights can be expressed as Eq. (4).

4 $$S\left( t \right) = f({\text{a}}_{1} \left( t \right){\text{,a}}_{2} \left( t \right)...{\text{a}}_{w} \left( t \right),\tau )$$

where $S\left( t \right)$ is the predicted fault state of the diagnostic system. $\tau$ represents the other parameters involved in this process.

Problem 4: To enhance the accuracy of diagnosis outcomes, optimization algorithms are essential for refining the initial parameters of the model. Due to the high complexity of the working environment of WSNs, experts cannot provide highly accurate information to construct the fault diagnosis model, so the initial parameters of the model provided by them are not optimal. Therefore, it is necessary to use parameter optimization algorithms to improve the parameters. This parameter optimization process can be denoted as Eq. (5).

5 $$\tau_{{{\text{best}}}} {\text{ = h}}\left( {\tau ,\beta } \right)$$

The optimized parameters are represented by $\tau_{{{\text{best}}}}$, where $\tau_{{}}$ denotes the parameters to be optimized. The optimization algorithm's parameters are indicated by $\beta$, and $h()$ represents the parameter optimization algorithm.

## Establishment of the WSN nodes fault diagnosis model based on BRB-AAW

The BRB-AAW technique is employed to address the main issues encountered during the fault diagnosis of WSN nodes in "Establishment of the WSN nodes fault diagnosis model based on BRB-AAW". This section will put forth resolutions to tackle these predicaments. The structure of the BRB-AAW-based WSN fault diagnosis model is described in "Basic structure of the model", the selection process of data features is recorded in "Extraction of data features", the modelling process of BRB-AAW is presented in "Formulation of rules and reasoning process of the BRB-AAW", the optimization process of model parameters by the P-CMA-ES algorithm is documented in "Optimization process of the model", and the overall modelling process of the BRB-AAW-based WSN fault diagnosis model is illustrated in "Model building process".

### Basic structure of the model

**Figure 1:**
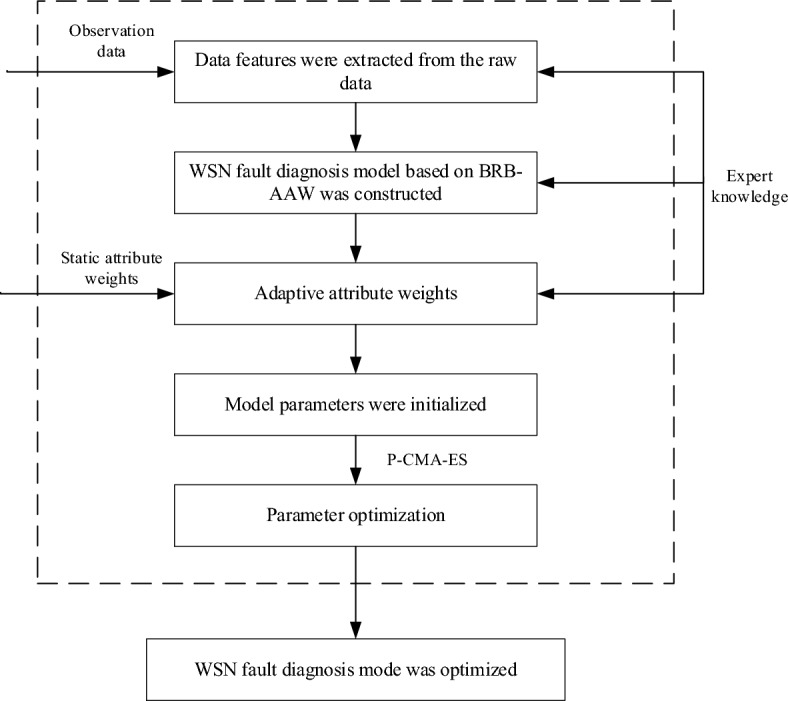
Elements of the BRB-AAW model.

The faults of the WSN nodes are diagnosed using the BRB-AAW approach, which involves breaking down the diagnosis model into four main components. First, data features that are beneficial for fault diagnosis are extracted from the original data. Second, the adaptive input attribute weights, initialized based on expert knowledge, are incorporated. The third component comprises the generation of rules and reasoning module, where the rules in BRB-AAW are initialized and the reasoning process is performed while considering the adaptive attribute weights. Finally, a constrained optimization algorithm is employed to refine the initial parameters of the model, in order to obtain more precise diagnosis outcomes. Figure 1 provides an illustration of the fundamental structure of the model.

### Extraction of data features

To distinguish the types of faults occurring in the nodes, various data features should be derived from the data collected by the WSN nodes. In the present study, MeanGap and Kurtosis are used as data features extracted to distinguish different types of faults^23^, with MeanGap helping to assess overall variability and Kurtosis highlighting extreme observations or critical thresholds in the dataset. In addition, both data features are time-varying and can be calculated using Eq. (6).

The MeanGap between moments $Q$ and $Q + T$ can be calculated using Eq. (6). $T$ indicates a time interval.

6 $$M{\text{ean}}G{\text{ap}}_{i} = \left| {\frac{{w\sum\limits_{t = Q}^{{Q{ + }T}} {{\text{a}}_{i} \left( t \right) - } \sum\limits_{t = Q}^{{Q{ + }T}} {\sum\limits_{j = 1}^{w} {{\text{a}}_{i} \left( t \right)} } }}{{w\left( {T{ + }1} \right)}}} \right|,i = 1,...,w$$

Kurtosis is a critical feature of the number distribution curve that characterizes the degree of sharpness or flatness of its peak. It can be calculated using Eq. (7).

7 $$Kurtosis_{i} = \frac{{\sum\limits_{t = Q}^{{Q{ + }T}} {\left[ {{\text{a}}_{i} \left( t \right) - \overline{{{\text{a}}_{i} }} } \right]^{4} } }}{{\left( {T{ + }1} \right)\upalpha ^{4} }}_{{}} i = 1,...,w$$

The average value of the data is represented by $\overline{{{\text{a}}_{i} }}$. The standard deviation of the data is denoted by $\upalpha ^{4}$.

### Formulation of rules and reasoning process of the BRB-AAW

After extracting the input data features, the next step is to use the data features as input attributes and then build the rules for the BRB-AAW model. The structure of the ${\text{kth}}$ rule $R_{k}$ is outlined in Eq. (8).

8 $$\begin{gathered} R_{k} :if{\kern 1pt} a_{1} \left( t \right){\kern 1pt} is{\kern 1pt} \, F_{1} {\kern 1pt} \cap {\kern 1pt} {\kern 1pt} a_{2} \left( t \right){\kern 1pt} is{\kern 1pt} \, F_{2} {\kern 1pt} \cap ...{\kern 1pt} \cap a_{W} \left( t \right){\kern 1pt} is{\kern 1pt} \, F_{W} {\kern 1pt} \, \hfill \\ Then{\kern 1pt} {\kern 1pt} {\kern 1pt} result{\kern 1pt} {\kern 1pt} is{\kern 1pt} \left\{ {\left( {H_{1} ,D_{1} } \right),\left( {H_{2} ,D_{2} } \right),...,\left( {H_{N} ,D_{N} } \right)} \right\} \hfill \\ with{\kern 1pt} {\kern 1pt} rule{\kern 1pt} {\kern 1pt} weight{\kern 1pt} {\kern 1pt} \theta_{k} \hfill \\ and{\kern 1pt} {\kern 1pt} attribute{\kern 1pt} {\kern 1pt} weight{\kern 1pt} {\kern 1pt} \delta_{1}^{k} ,\delta_{2}^{k} ,...,\delta_{w}^{k} \hfill \\ \end{gathered}$$

The model has ${\text{w}}$ input attributes denoted by ${\text{a}}_{{\text{i}}} (t)(i = 1,2...,w)$, and the set of reference values used to evaluate these attributes is represented by $F_{{\text{i}}} (i = 1,2...,w)$. The BRB-AAW model produces $N$ fault state evaluation results, denoted by $H_{{\text{i}}} (i = 1,2...,N)$. Each of these results corresponds to a belief degree $\theta_{{\text{i}}} (i = 1,2...,k)$ under the $k{\text{th}}$ belief rule. The $i - th$ belief rule has a rule weight of $\theta_{i}$, and there are ${\text{k}}$ such belief rules. Additionally, $\delta_{1}^{{\text{k}}} ,...,\delta_{W}^{{\text{k}}}$ represents the adaptive attribute weights in the $i - th$ belief rule in the set of adaptive attribute weights $\left[ {\updelta _{1}^{1} {,}\updelta _{2}^{1} ,...,\updelta _{w}^{1} } \right],...,\left[ {\updelta _{1}^{k} {,}\updelta _{2}^{k} ,...,\updelta _{w}^{k} } \right]$, which is obtained based on the simulation judgment results of data features and expert knowledge. Adaptive attribute weights can avoid the influence of unreliable data features in different environments.

After completing the rule construction process, the next step is to incorporate the adaptive attribute weights into the inference process of the BRB model. This study's BRB model inference process, considering adaptive attribute weights, consists of five distinct steps.

Step 1: The initial step in the inference process involves calculating the matching degree of attributes. This is accomplished by inputting attribute data and calculating the corresponding matching degree with reference points, utilizing input and reference values as a basis. The calculation process is described by Eq. (9).

9 $$\uprho _{{\text{i}}}^{{\text{j}}} { = }\left\{ \begin{gathered} \frac{{F_{{\text{i}}}^{{\text{k + 1}}} - a_{i} \left( {\text{t}} \right)}}{{{\text{F}}_{{\text{i}}}^{{\text{k + 1}}} - {\text{F}}_{{\text{i}}}^{{\text{k}}} }},j = K \hfill \\ \frac{{a_{i} \left( {\text{t}} \right) - {\text{F}}_{{\text{i}}}^{{\text{k}}} }}{{{\text{F}}_{{\text{i}}}^{{\text{k + 1}}} - {\text{F}}_{{\text{i}}}^{{\text{k}}} }},j = K{ + 1} \hfill \\ {\text{0,j = 1,2,}}...{\text{,K,j}} \ne {\text{K,j}} \ne {\text{K}} + 1 \hfill \\ \end{gathered} \right.$$

The equation encompasses the matching degree of the $j{\text{th}}$ reference value of the $i{\text{th}}$ attribute, denoted as $\uprho _{{\text{i}}}^{{\text{j}}}$, the current value of the input attribute denoted by $a_{j}$, and the $k{\text{th}}$ reference value of the $i{\text{th}}$ attribute denoted by $F_{{\text{i}}}^{{\text{k}}}$. This formula can be utilized to compute the degree of match of the attribute reference value when the condition represented by ${\text{F}}_{{\text{i}}}^{{\text{k}}} \le a_{i} \left( {\text{t}} \right) \le {\text{F}}_{{\text{i}}}^{{\text{k + 1}}}$ is fulfilled.

Step 2: Initialization of adaptive attribute weights is considered. In this step, the fixed attribute weights are initialized to different adaptive attribute weights based on the initialization information provided by expert knowledge^24^. The initialization process is shown in Eq. (3):

Step 3: The matching degree of the $k{\text{th}}$ rule is computed by computing the attribute matching. Subsequently, the match of the rule in BRB needs to be determined. If the match of the rule is nonzero, the rule is activated; otherwise, it remains deactivated. Equation (10) illustrates the formula for calculating the matching degree of a rule.$\rho_{{\text{k}}}^{{\text{i}}}$ represents the matching degree of the $i{\text{th}}$ attribute in the $k{\text{th}}$ rule.

10 $$\uprho _{k} = \prod\limits_{i = 1}^{W} {\left( {\uprho _{k}^{i} } \right)}$$

Step 4: Once the matching degree of the rules has been calculated and the activated rules have been identified, the next step is to calculate the activation weights of the rules. The activation weights can be computed using Eq. (11).

11 $$\mho_{i} = \frac{1}{{\sum\limits_{j = 1}^{K} {\theta_{j}\uprho _{j} } }}\theta_{i}\uprho _{i} ,i = 1,2,...,K$$

In the equation above, the variable $\theta_{i}$ represents the weight of the $k{\text{th}}$ rule, while the variable $\rho_{i}$ represents the matching degree of the same rule. The symbol $K$ represents the total number of rules in the BRB model. Only the rules that are activated have a nonzero activation weight, while the activation weight for nonactivated rules is zero.

Step 5: After identifying the activated rules, they are integrated using an ER algorithm to obtain a fused belief degree^25^. The calculation method for this is demonstrated through Eqs. (13) and (14).

13 $$\psi = \left[ {\sum\limits_{n = 1}^{N} {\prod\limits_{i = 1}^{K} {\left( {\mho_{i} D_{n,i} + 1 - \mho_{i} \sum\limits_{j = 1}^{N} {D_{j,i} } } \right)} - \left( {N - 1} \right)} \prod\limits_{i = 1}^{N} {\left( {1 - \mho_{i} \sum\limits_{j = 1}^{N} {D_{j,i} } } \right)} } \right]^{ - 1}$$

14 $$D_{n} = \psi \frac{{\left[ {\prod\limits_{i = 1}^{K} {\left( {\mho_{i} D_{n,i} + 1 - \mho_{i} \sum\limits_{j = 1}^{N} {D_{j,i} } } \right) - } \prod\limits_{i = 1}^{K} {\left( {1 - \mho_{i} \sum\limits_{j = 1}^{N} {D_{j,i} } } \right)} } \right]}}{{1 - \psi \prod\limits_{i = 1}^{K} {\left( {1 - \mho_{i} \sum\limits_{j = 1}^{N} {D_{j,i} } } \right)} }}$$

The framework for identifying fault diagnosis models consists of $N$ levels, denoted as $N$, while the number of rules that have been triggered is represented by $K$. The activation weight of a rule is denoted by $\mho_{i}$, while the belief degree of consequent $j$ in rule $i$ is represented by $D_{j,i}$. The expert knowledge determines the initial belief degree. By using Eqs. (12) and (13) for calculation, Eq. (14) shows the resulting output.

14 $$S\left( {\text{t}} \right) = \sum\limits_{i = 1}^{N} {p\left( {H_{i} } \right)D_{i} }$$

where $p\left( {H_{i} } \right)$ is the utility of the $i{\text{th}}$ fault diagnosis, determined by the expert based on the actual fault state. $S\left( {\text{t}} \right)$ represents the final diagnosis result obtained by the fault diagnosis model.

**Figure 2:**
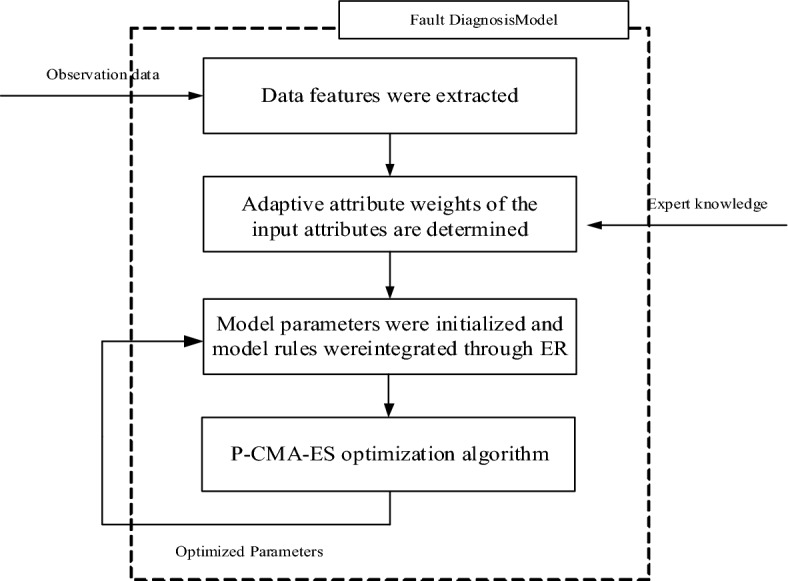
Reasoning process of the BRB-AAW.

The above analysis presents an introduction to the inference process of BRB-AAW. Figure 2 illustrates the entire inference process in graphical format.

### Optimization process of the model

The initial values for rule weights, belief degrees of BRB-AAW, and adaptive attribute weights are determined by expert knowledge, as explained in "Extraction of data features". However, in cases where the model comprises a large number of parameters and the experts' experience and knowledge are insufficient, the initial parameter settings may not be reasonable. This could have an adverse impact on the model diagnosis accuracy. To address this issue, this paper proposes a model optimization process that employs the projection covariance matrix adaptive evolution strategy (P-CMA-ES) to optimize the model parameters^26,27^. The optimized parameters that require optimization must satisfy the following conditions.

15 $$\begin{gathered} \min MSE(\tau ) \hfill \\ s.t. \hfill \\ \sum\limits_{{{\text{n}} = 1}}^{N} {D_{{\text{n,i}}} } \le 1,i = 1,2,...,K \hfill \\ 0 \le D_{{\text{n,i}}} \le 1,n = 1,2,...,N,i = 1,2,...,W \hfill \\ 0 \le \theta_{{\text{i}}} \le 1,i = 1,2,...,K \hfill \\ 0 \le \delta_{i}^{j} \le 1,i = 1,2,...,W,j = 1,2,...,K \hfill \\ \end{gathered}$$

To optimize the model, the objective function employed is the mean square error (MSE), represented by MSE. Equation (15) provides the expression for this objective function.

**Figure 3:**
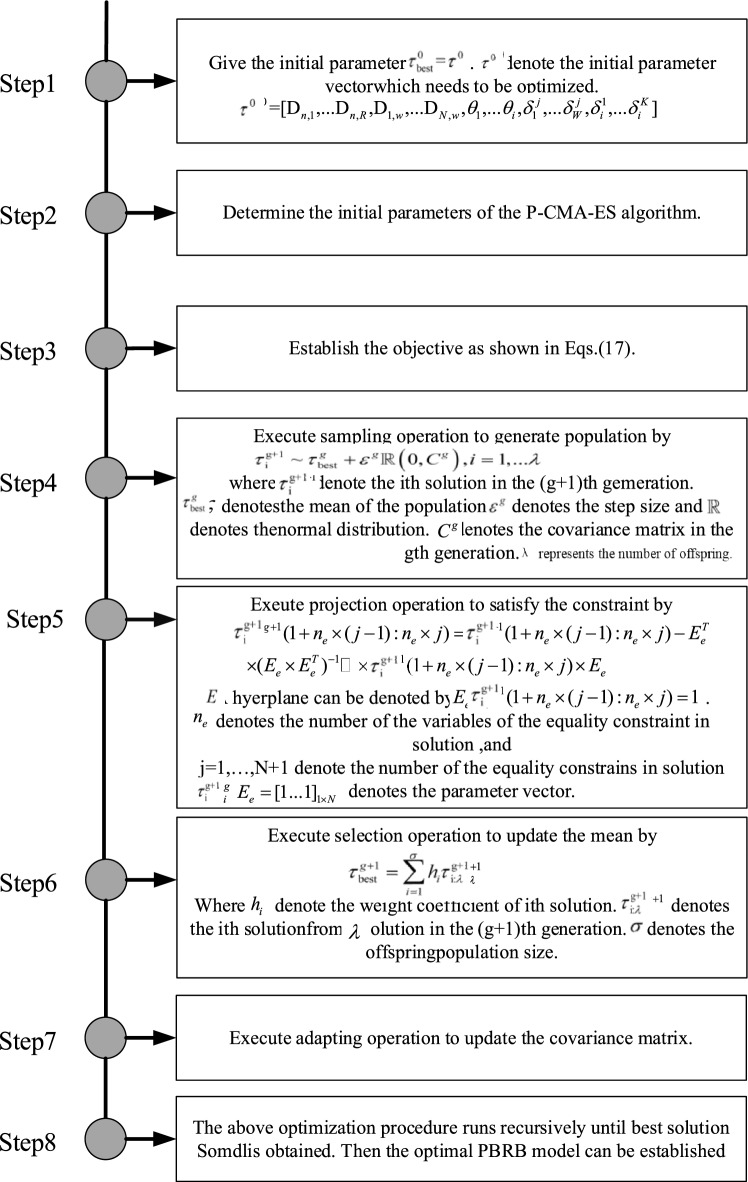
Optimization process using the P-CMA-ES algorithm.

Once the parameters to be fine-tuned and the constraints have been established, it is necessary to define a metric that reflects the efficacy of the optimization. Suppose that the outcome of the optimization procedure is $S({\text{t}})_{{{\text{expected}}}}^{{}}$, whereas the initial output from the dataset for training purposes is $S(t)_{{}}^{{}}$. Based on the analysis above, the optimization process of the model can be interpreted as the quest for the minimum threshold of the mean square error (MSE) between the predicted results and the actual results. The MSE is calculated using Eq. (16), where NUM signifies the count of data points employed in the optimization of the parameters. The optimization of model parameters is carried out using the P-CMA-ES algorithm, which is illustrated in Fig. 3.

16 $$MSE(\tau_{{}} ) = \frac{{\sum\limits_{{{\text{i}} = 1}}^{NUM} {\left( {S(t)_{{{\text{expected}}}}^{{}} - S(t)_{{}}^{{}} } \right)^{2} } }}{NUM}$$

### Model building process

**Figure 4:**
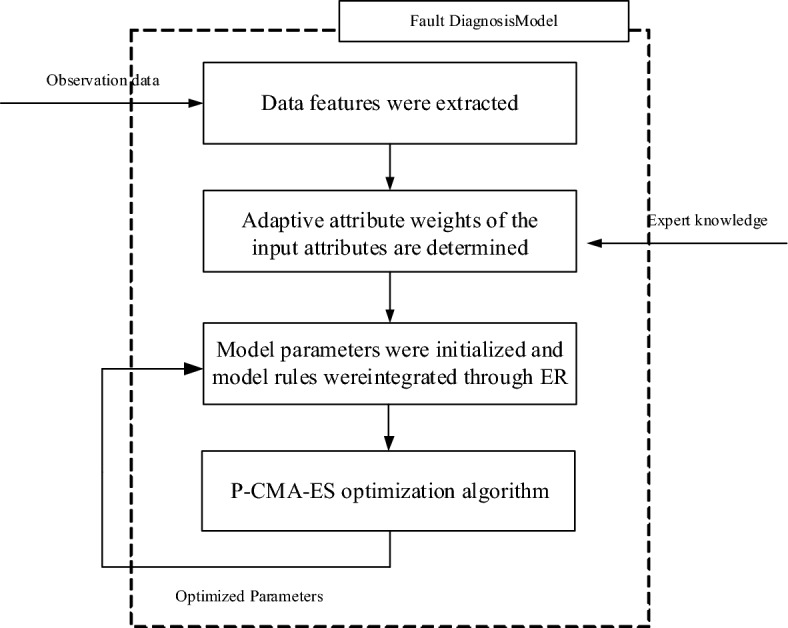
BRB-AAW-based fault diagnosis model for WSN nodes.

Upon analysing the contents of this section, the process of creating a WSN node diagnostic fault model using BRB-AAW can be broken down into the following steps, as shown in Fig. 4:

Step 1: Extract the WSN node's data features from the original data and employ them as the initial attributes of the BRB-AAW model.

Step 2: Improve the static attribute weights and obtain reasonable attribute weights based on expert knowledge.

Step 3: The parameters are given initial values, and rules are integrated by the ER parsing algorithm.

Step 4: To enhance the diagnostic accuracy of the model, the initial parameters are optimized using the P-CMA-ES algorithm.

## Case study

**Figure 5:**
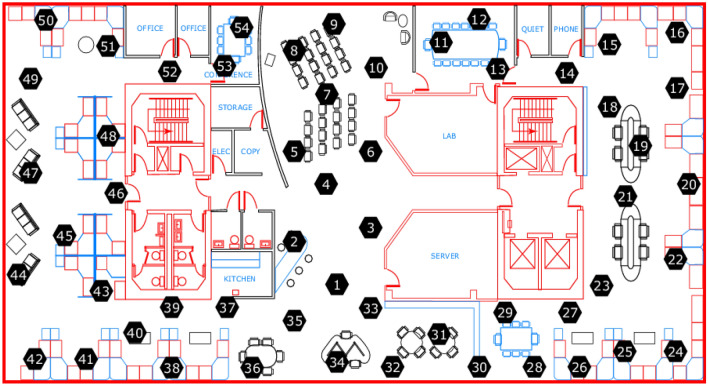
Distribution of laboratory sensors.

Within this section, a comparative analysis is performed between BRB-AAW and other fault diagnosis methods using sensor data sourced from Intel Berkeley Research Lab. The findings demonstrate a notable enhancement in the accuracy of fault diagnosis results for WSN nodes when utilizing the BRB-AAW method. The sensor distribution is shown in Fig. 5. The necessary information for the Intel Labs data is shown in Table 2. The process of obtaining the observed data and the fault identification framework setup is formulated in "Dataset setting", the initial parameters needed for the BRB-AAW model are calculated in "Construction of the BRB-AAW model", the metrics observed in the example are documented in "Training and testing of the model", the results of the comparisons between the parameter optimization algorithms are shown in "Comparison of optimization algorithms", the results of the comparative experiments with multiple metrics are shown in "Results of the experiment", and the comparison of the BRB and the BRB-AAW with the multiple rounds of the control variable experiments are documented in "Comparison with other methods".

**Table 2:** Information about the dataset.

Parameter	Information
Acquisition time	February 28, 2004—April 5, 2004
Collection location	The Intel Berkeley Research lab
Sensor type	Mica2Dot sensors with weatherboards
Data acquisition system	TinyDB5431
Number of sensors	54
Acquisition interval	31 s
Collecting information	Temperature, humidity, light, and voltage
Data size	2.3 million

### Dataset setting

Step 1: The dataset undergoes preprocessing to establish the diagnostic result set of the model. It comprises thermal conditions, atmospheric moisture, brightness, and electrical potential data gathered by sensors deployed within a laboratory room. Taking into account the sensor arrangement and the trajectory of the data ^28^, temperature readings collected by sensors 1 through 4 recorded between March 1 and 7 were selected for further analysis. Preprocessing is necessary due to data gaps. Furthermore, to showcase the model's capacity to effectively address noisy data, Gaussian noise is added during dataset simulation.

**Figure 6:**
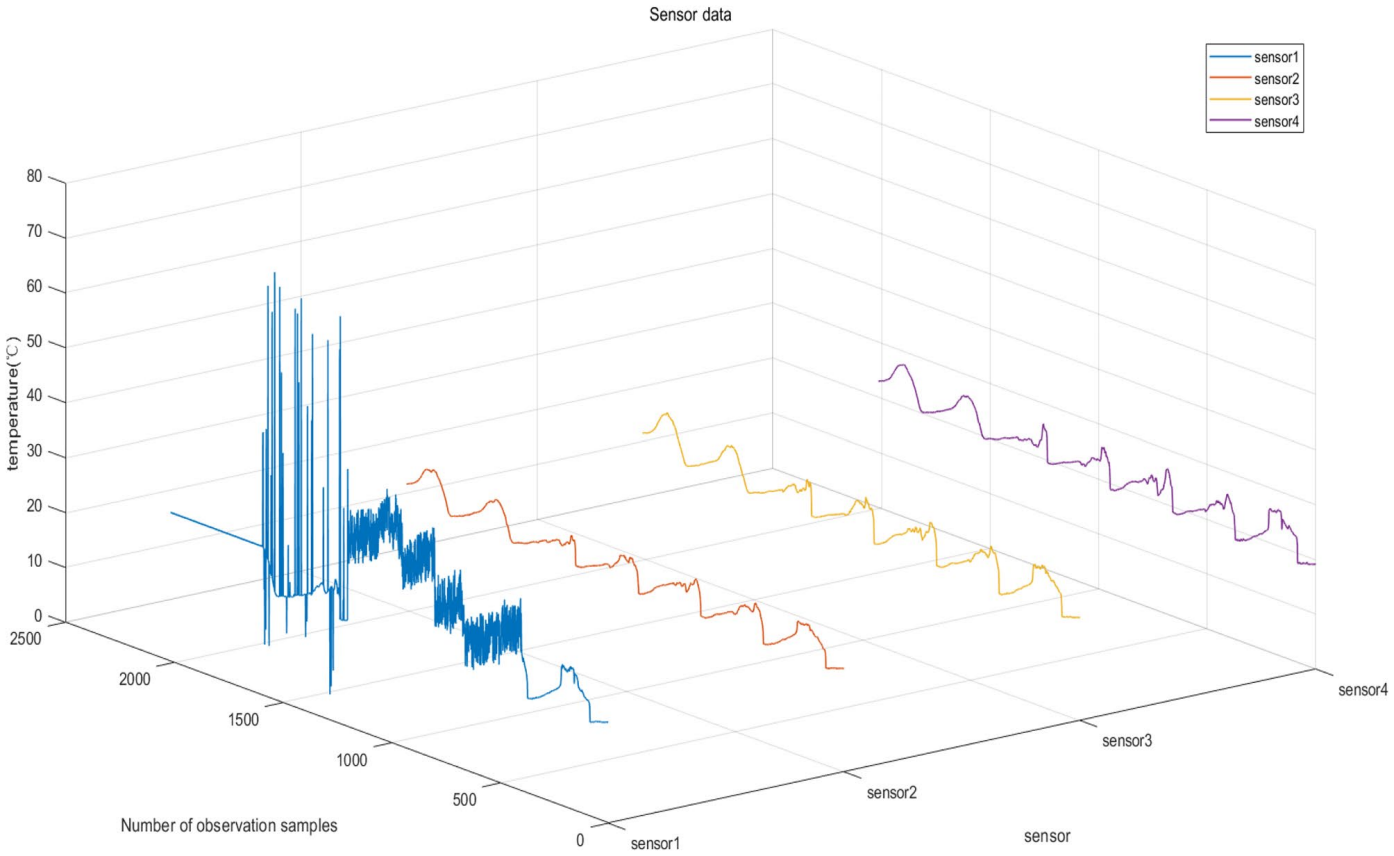
Sensor data after preprocessing.

After processing, the dataset comprises a total of 2016 data points with a 5-min interval between successive records. Figure 6 illustrates the temperature data recorded by sensors 1 through 4.

**Figure 7:**
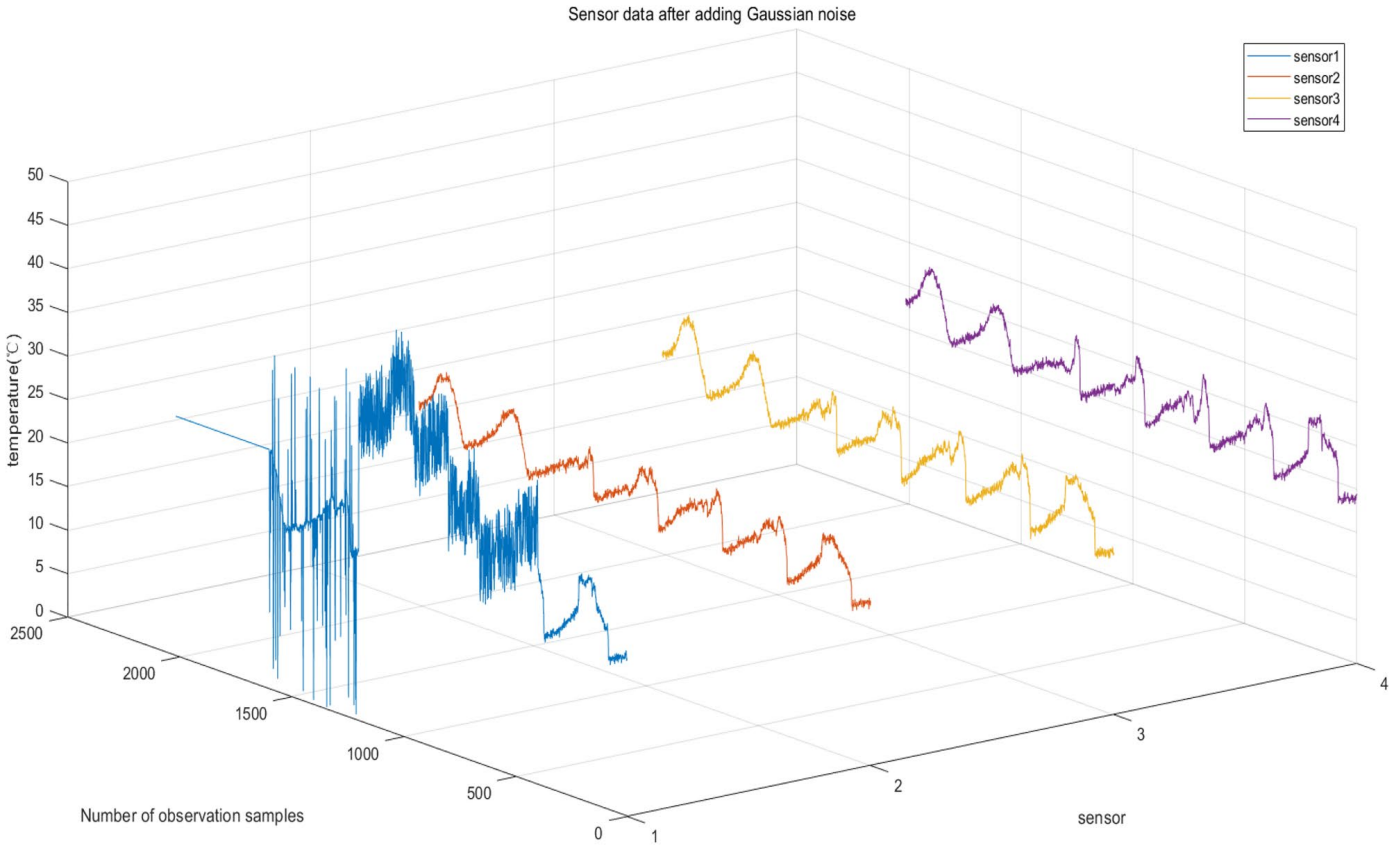
Sensor data after adding Gaussian noise.

Step 2: This paper endeavors to identify sensor states, including normal, fixed-value faults, outlier faults, high-noise faults, and offset faults. To achieve this, the simulation of the aforementioned five sensor state types on sensor 1 was carried out using a software-based approach that aligns with specific characteristics of faults. The technique employed for simulating state data is presented in Table 3, and Fig. 7 illustrates the resulting simulated state data. In Fig. 7, 1–399 indicates normal data, and 400–799 indicates offset fault data. 800–1199 is high noise fault data, 1200–1599 is abnormal value fault data and 1600–2016 is fixed value fault data.

**Table 3:** Fault simulation methods.

Fault type	Simulation Method
Offset faults	Random numbers within the range of [0, 10] were added to the data between sample numbers 400 and 799 by means of superimposition
High noise faults	The dataset consisting of sample numbers 800 to 1199 is created by adding random numbers from the range of [10, 20]
Outlier faults	A random number generator was used to replace 10% of the data in the sample range of 1200 to 1599 with values in the interval of [0, 40]
Fixed faults	The data collected is kept constant at the value measured immediately prior to the occurrence of the fault

Step 3: According to the dataset in the case study, the framework for identifying fault diagnosis models produces five separate states: normal states (NS), offset faults (OSF), high noise faults (HNF), outlier faults (OLF), and fixed value faults (FVF). These states are defined by Eq. (17), with their respective reference values provided in Eq. (18).

17 $$\{ NS,OSF,HNF,OLF,FVF\}$$

18 $$\{ 0,1,2,3,4\}$$

### Construction of the BRB-AAW model

**Figure 8:**
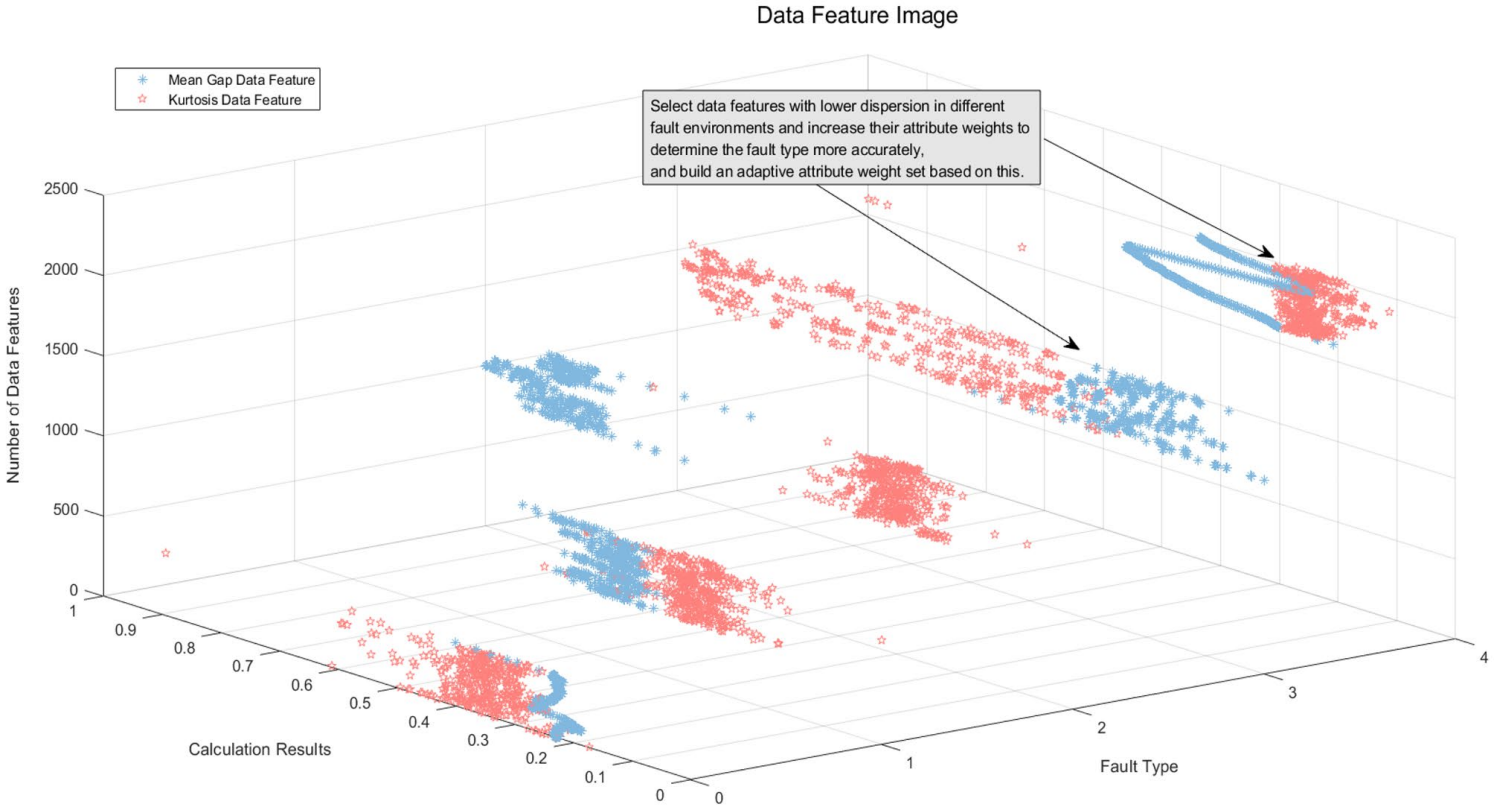
Mean gap data feature image.

Step 1: Following data preparation, the data features were extracted utilizing the approach outlined in "Dataset setting". Specifically, the $MeanGap$ and $Kurtosis$ were extracted as input features of the model. The duration for feature extraction was set to 12, and the resulting features were normalized. Figure 8 illustrates the extracted data features. The mean gap data feature in the 800–1199 part of Fig. 8 is more effective in distinguishing between differentiated fault types than the $Kurtosis$ data feature in the same part of Fig. 8. Therefore, this serves as an important basis for expert knowledge to provide the set of adaptive attribute weights.

**Figure 9:**
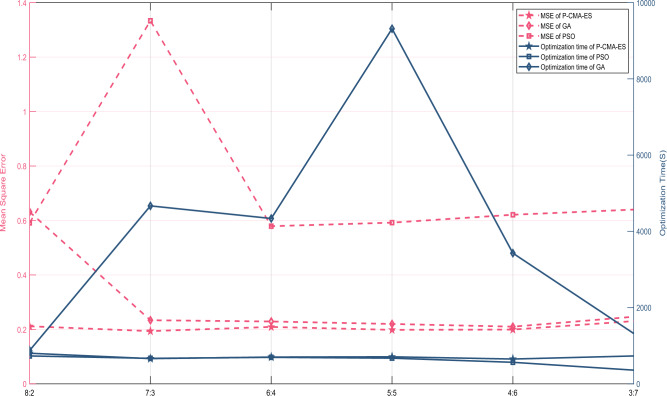
Comparative results of optimization algorithms.

Step 2: After the extraction of data features, it is necessary to determine the reference values and reference points for each attribute. To accomplish this, the data distribution characteristics of Figs. 8 and 9 were considered. Specifically, benchmark values and reference points for the MeanGap and Kurtosis were established. For the MeanGap, reference points were established as small (S), relatively small (RS), medium (M), relatively large (RL), and large (L), with corresponding reference values presented in Table 4. Conversely, *Kurtosis* was characterized by reference points of small (S), relatively small (RS), relatively large (RL), and large (L), with associated reference values displayed in Table 5.

**Table 4:** Reference value of mean gap.

Reference point	S	RS	M	RL	L
Reference value	−0.001	0.184	0.358	0.700	1.001

**Table 5:** Reference value of kurtosis.

Reference point	S	RS	RL	L
Reference value	−0.001	0.184	0.700	1.001

Step 3: The optimization of other parameters of the model is performed by applying the P-CMA-ES approach described in "Optimization process of the model". The optimized parameters are presented in Table 6, where each row corresponds to a rule. After optimization, the model inference process described in "Formulation of rules and reasoning process of the BRB-AAW" is employed in conjunction with the optimized parameters for the purpose of sensor fault diagnosis and result generation.

**Table 6:** Tolerance range parameters of data features.

Input attributes	Diagnostic results	Average value	Standard deviation
Mean gap	NS	0.2333	0.0213
	OSF	0.4633	0.0405
	HNF	0.8723	0.0563
	OLF	0.2236	0.0702
	FVF	0.4071	0.0987
Kurtosis	NS	0.1715	0.1084
	OSF	0.1085	0.0571
	HNF	0.1018	0.0519
	OLF	0.5866	0.2924
	FVF	0.0090	0.0917

### Training and testing of the model

Training data and iterative data are determined, and data features are extracted from the simulated fault data. The data samples are randomly divided into 3:7, 4:6, 5:5, 6:4, 7:3, and 8:2 according to the commonly used training and test set ratios.

The evaluation criteria for the fault diagnosis model are established. To assess the effectiveness of the method, the evaluation metrics chosen include overall accuracy, false negative rate (FNR), false positive rate (FPR), true positive rate (TPR), true negative rate (TNR), and precision. In this context, samples without faults are considered negative samples, while samples with faults are considered positive samples. The equations representing these metrics are provided as follows: Eq.  (19) for $Accuracy$, Eq. (20) for $FNR$, Eq. (21) for $FPR$, Eq. (22) for $TNR$, Eq. (23) for $TPR$, and Eq. (24) for $Precision$.

19 $$A{\text{ccuracy = }}\frac{{NUM_{{{\text{right}}}} }}{{NUM_{{{\text{brochure}}}} }}$$

The variable $NUM_{{{\text{brochure}}}}$ indicates the quantity of correctly diagnosed samples by the model. The term $NUM_{{{\text{right}}}}$ refers to the overall number of samples analysed.

20 $$FNR = \frac{FN}{{TP + FN}}$$

The variable FN represents the quantity of samples that were falsely diagnosed as negative, while TP indicates the number of samples that were accurately diagnosed as positive.

21 $$FPR = \frac{FP}{{FP + TN}}$$

The variable FP denotes the quantity of samples that were falsely diagnosed as positive, while TN indicates the number of samples that were correctly diagnosed as negative.

22 $$TNR = \frac{TN}{{TN + FP}}$$

The variable TN denotes the quantity of samples judged negative and actually negative.

23 $$TPR = \frac{TP}{{TP + FN}}$$

The variable TP denotes the quantity of samples judged positive and actually positive, while TN indicates the number of samples that were correctly diagnosed as negative.

24 $$\Pr {\text{ecision}} = \frac{TP}{{TP + FP}}$$

### Comparison of optimization algorithms

Expert knowledge is utilized to set parameters such as adaptive attribute weights and rule weights, but these initial parameter settings may not be the best. Therefore, optimization algorithms are employed to achieve more precise diagnosis results. Three common optimization algorithms for BRB, namely, particle swarm optimization (PSO), the genetic algorithm (GA), and the P-CMA-ES method, are tested on six distinct datasets of varying sizes, as described in "Training and testing of the model". The test outcomes are illustrated in Fig. 9. First, the GA exhibits the highest MSE value and the least favorable optimization effect. The PSO algorithm and P-CMA-ES algorithm yield similar MSE values. Second, in terms of optimization time, the GA algorithm and P-CMA-ES algorithm demonstrate comparable performance. However, the GA exhibits an excessively high MSE value. Finally, the P-CMA-ES algorithm emerges as the most effective optimization algorithm for the model. The optimized results are presented in Table 7.

**Table 7:** Optimized model parameters.

Ruler number	Input attributes		Rule weight	Belief distribution
	MeanGap	Kurtosis		{NS, OSF, HNF, OLF, FVF}
1	S	S	0.16930.3636	0.21816, 0.09817, 0.27579, 0.13844, 0.26944
2	S	RS	0.4936	0.0024, 0.40539, 0.01229, 0.03631, 0.54362
3	S	RL	0.9784	0.0623, 0.06429, 0.06813, 0.41309, 0.39219
4	S	L	0.9234	0.09458, 0.135282, 0.065474, 0.12399, 0.580676
5	RS	S	0.0062	0.0448, 0.517374, 0.00352, 0.000652, 0.433654
6	RS	RS	0.0118	0.99599, 0.00193, 0.00006, 0.00172, 0.00031
7	RS	RL	0.0028	0.36190, 0.36689, 0.25268, 0.00607, 0.01246
8	RS	L	0.0002	0.30607, 0.07011, 0.12829, 0.10386, 0.39168
9	M	S	0.8700	0.00050, 0.00036, 0.00189, 0.00124, 0.99601
10	M	RS	0.0001	0.63492, 0.05458, 0.09995, 0.15465, 0.05589
11	M	RL	0.0033	0.22425, 0.07192, 0.08152, 0.26334, 0.35897
12	M	L	0.0082	0.02146, 0.17697, 0.07801, 0.11676, 0.60680
13	RL	S	0.0094	0.88110, 0.07109, 0.04148, 0.00366, 0.00267
14	RL	RS	0.0723	0.45348, 0.30286, 0.03648, 0.01569, 0.19148
15	RL	RL	0.2681	0.47403, 0.19242, 0.21118, 0.0733, 0.04904
16	RL	L	0.0964	0.21497, 0.18619, 0.08708, 0.38059, 0.13118
17	L	S	0.7690	0.00430, 0.09177, 0.34379, 0.52273, 0.03741
18	L	RS	0.9867	0.00018, 0.56913, 0.11641, 0.03250, 0.28178
19	L	RL	0.9347	0.08987, 0.35595, 0.10905, 0.13588, 0.30925
20	L	L	0.3636	0.08792, 0.17882, 0.23044, 0.05537, 0.44745

### Results of the experiment

**Figure 10:**
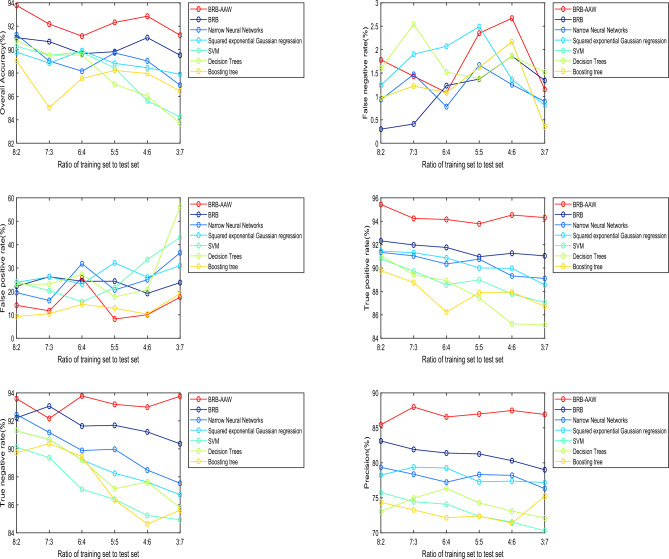
Experimental results for different ratios of training samples to test samples.

To verify that the BRB-AAW-based WSN node fault diagnosis method can effectively reduce the influence of noisy data on the WSN node fault diagnosis process, experiments were conducted using BRB-AAW, artificial neural network, Gaussian regression, SVM, decision tree, and boosting tree at different ratios of training samples to test samples. In addition to the above methods, it can be compared with algorithms such as logistic regression, plain Bayesian classifier, K-nearest neighbor classification, and K-mean clustering, and the comparison metrics include the overall accuracy, false negative rate (FNR), false positive rate (FPR), true positive rate (TPR), true negative rate (TNR), and precision. The experimental results are presented in Fig. 10. Indicators data for different methods at different ratios of training samples to test samples can be seen in Table 8.

**Table 8:** Indicators data for different methods at different ratios of training samples to test samples.

indicators	Methods	8:2 (%)	7:3 (%)	6:4 (%)	5:5 (%)	4:6 (%)	3:7 (%)	Average (%)
Overall accuracy	BRB-AAW	93.77	92.18	91.15	92.32	92.85	91.23	92.25
	BRB	91.02	90.68	89.65	89.82	91.02	89.52	90.29
	Narrow neural networks	91.27	89.02	88.15	89.72	89.03	86.96	89.02
	Squared exponential Gaussian regression	89.78	88.85	89.90	88.82	88.45	87.88	88.95
	SVM	90.27	89.52	89.78	88.42	85.62	84.25	87.98
	Decision trees	90.77	89.52	89.53	87.03	86.03	83.68	87.76
	Boosting tree	89.03	85.04	87.53	88.22	87.95	86.46	87.37
False negative rate	BRB-AAW	1.78	1.43	1.08	2.35	2.67	1.15	1.74
	BRB	0.30	0.41	1.23	1.37	1.86	1.34	1.09
	Narrow neural networks	0.93	1.47	0.78	1.67	1.25	0.89	1.16
	Squared exponential Gaussian regression	0.64	1.67	1.57	0.63	1.68	0.89	1.18
	SVM	1.24	1.90	2.07	2.49	1.36	0.81	1.65
	Decision trees	0.97	1.22	1.08	1.62	2.17	0.35	1.23
	Boosting tree	1.59	2.54	1.52	1.38	1.86	1.52	1.73
False positive rate	BRB-AAW	14.06	11.71	25.83	8.21	10.04	17.54	14.56
	BRB	22.22	26.27	24.34	24.37	19.15	23.76	23.35
	Narrow neural networks	19.48	16.13	31.85	20.63	25	36.50	24.93
	Squared exponential Gaussian regression	23.86	26.23	23.17	32.20	26.09	31.05	27.10
	SVM	24.05	20.31	15.61	21.61	33.60	43.10	26.38
	Decision trees	23.08	23.15	27.27	17.68	20.68	56.18	28.00
	Boosting tree	9.30	10.47	14.48	12.81	10.30	19.08	12.74
True positive rate	BRB-AAW	95.43	94.24	94.05	93.78	94.53	94.31	94.39
	BRB	92.32	91.97	91.76	90.97	91.26	91.04	91.55
	Narrow neural networks	91.45	91.31	90.87	90.01	89.97	88.56	90.36
	Squared exponential Gaussian regression	90.79	89.73	88.56	88.99	87.78	87.06	88.82
	SVM	91.07	89.43	88.92	87.45	85.23	85.14	87.87
	Decision trees	90.08	89.32	88.76	86.43	84.76	83.35	87.12
	Boosting tree	89.78	88.76	86.23	87.89	87.94	86.73	73.07
True negative rate	BRB-AAW	93.78	92.07	94.11	93.92	93.87	93.99	93.62
	BRB	92.14	93.12	91.89	91.76	91.38	90.83	91.85
	Narrow neural networks	92.18	91.73	90.60	90.32	89.21	88.18	90.37
	Squared exponential Gaussian regression	91.32	91.03	89.12	87.13	87.98	87.37	88.99
	SVM	90.78	89.77	87.99	87.21	85.39	85.02	87.69
	Decision trees	91.98	91.20	89.93	87.92	88.08	86.68	89.30
	Boosting tree	89.39	90.82	89.73	87.12	84.46	85.08	87.77
Precision	BRB-AAW	85.78	87.07	85.91	86.72	86.97	86.69	86.52
	BRB	83.14	82.92	82.39	82.33	81.98	80.73	82.25
	Narrow neural networks	79.28	78.93	78.62	79.22	79.27	77.12	78.74
	Squared exponential Gaussian regression	78.22	79.27	79.32	77.98	78.02	78.17	78.50
	SVM	76.28	75.60	74.92	73.93	72.79	70.07	73.93
	Decision trees	72.53	75.27	77.26	74.91	74.18	72.88	74.51
	Boosting tree	73.69	73.02	72.73	72.82	72.36	75.87	73.42

### Comparison with other methods

**Figure 11:**
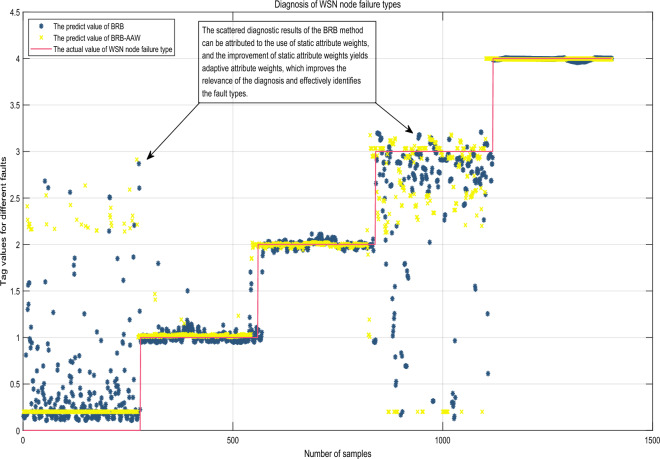
Evaluation of the diagnostic results of the BRB-AAW in comparison to the BRB.

**Figure 12:**
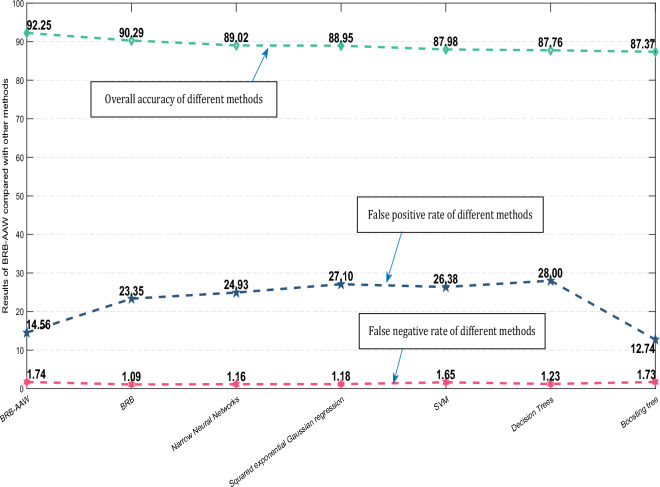
The average results of BRB-AAW compared with other methods.

**Figure 13:**
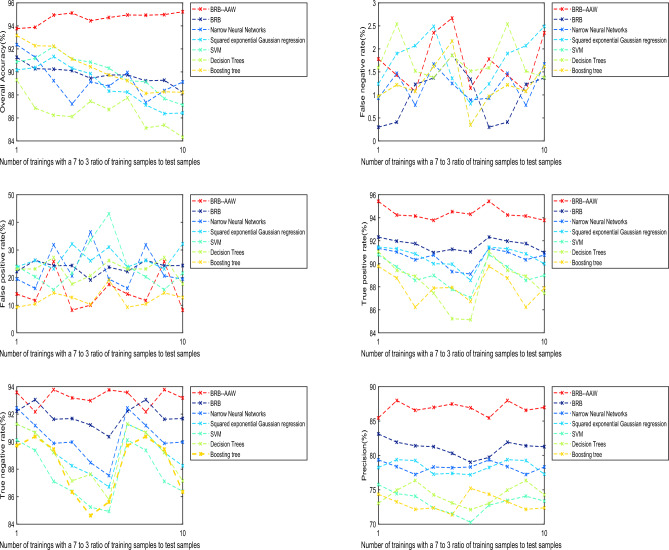
All results of BRB-AAW compared to other methods at the same training to test sample ratio.

To assess the effectiveness of the method proposed in this paper and the advancements made by the BRB method, a comparison with other methods was carried out. The fault diagnosis results were compared to those obtained using the BRB method, as illustrated in Fig. 11. The average values of the assessment indicators for each method are presented in Fig. 12. Taking the ratio of training samples to test samples as 7 to 3 as an example, the values of all assessment metrics of each method are shown in Fig. 13.

Upon conducting a comparison between the outcomes of the BRB-AAW and BRB, it becomes apparent that the BRB-AAW values more accurately reflect the real scenario, particularly when the standard value is 0. The test results indicated that BRB-AAW's overall accuracy, narrow neural networks, and SVM were 92.25%, 90.29%, and 87.98%, respectively, while the corresponding values for BRB were 90.24%, 23.35%, and 1.09%^29^. The scattered diagnostic results of the BRB approach can be attributed to the use of static attribute weights, which can lead to incorrect fault type identification based on attribute weights. To address this issue, adaptive attribute weights are obtained by improving static attribute weights, which enhances the relevance of diagnosis and effectively identifies fault types.

Based on the comparison of evaluation indexes among different methods, the following conclusions can be drawn. First, the BRB-AAW and BRB methods generally outperform other approaches in relation to $Accuracy$. Second, the BRB-AAW method exhibits a low occurrence of false positives, which reduces the likelihood of nodes being misdiagnosed as faults. Finally, there are minor discrepancies in the false-negative rate across all methods, fluctuating by approximately 1.3% or less, and there are no significant differences observed.

These results can be attributed to several factors. First, the BRB-AAW method incorporates adaptive attribute weights and enhances the calculation methodology for attribute weights, enabling the identification of reliable fault types and more precise adaptive attribute weights. Second, the reasoning approach employed by BRB-AAW is similar to that of BRB and can effectively deal with ambiguous information, including vagueness, unpredictability, and ignorance. Finally, the configuration of the model parameters for BRB-AAW is based on expert knowledge and experience, resulting in more reasonable parameter values. The synergistic effect of these factors contributes to the improved accuracy of diagnosis in the BRB-AAW method.

## Conclusion

After analysing the relevant literature, there are three problems with the current fault diagnosis methods used for WSNs. First, these methods do not take into account the effect of environmental noise on sensor readings during fault diagnosis. Second, the neural network-based methods require a large number of uniform fault samples to train the model parameters, which makes them less practical. Finally, the attribute reliability calculation of BRB methods cannot consider unreliable data with large variations in attribute values in the middle section, which leads to inaccurate fault diagnosis. To address these drawbacks, in this paper, the BRB-AAW model is proposed as a fault diagnosis method.

Nevertheless, the BRB-AAW method has some limitations. Future research will focus on solving the combinational explosion problem in the BRB-AAW model by reducing the number of rules. A network structure with a BRB will also be designed to minimize the number of rules per submodule and help parameter initialization. In addition, the construction method of the BRB network structure will be explored and designed to enhance its model consistency with the underlying working mechanism.

## Data Availability

The datasets used during the current study are available from the corresponding author upon reasonable request.
